# The impact of mixed-anonymity on brainstorming performance: Evidence from field experiments

**DOI:** 10.1371/journal.pone.0354236

**Published:** 2026-07-21

**Authors:** Theresa Wöllner, Tim G. Schweisfurth, Alexander Brem

**Affiliations:** 1 Institute of Entrepreneurship and Innovation Science, University of Stuttgart, Stuttgart, Germany; 2 Institute for Organizational Design and Collaboration Engineering, Hamburg University of Technology, Hamburg, Germany; 3 Department of Technology and Innovation, University of Southern Denmark, Sønderborg, Denmark; Vanderbilt University, UNITED STATES OF AMERICA

## Abstract

Generating innovative and valuable ideas is essential for organizational competitiveness, and structured ideation formats are widely used to support creativity and idea generation of individuals, teams, and organizations. One such format is nominal brainstorming, in which participants generate ideas independently rather than through group interactions. Previous research has produced mixed results regarding the impact of anonymity in such individual idea-generation settings. Anonymous formats may reduce evaluation apprehension but can also lead to free riding, while non-anonymous formats promote accountability yet might increase the fear of negative evaluation. To clarify these conflicting findings, this study examines a novel mixed-anonymity approach in which participants generate ideas independently and anonymously, but the identities of those with the highest-rated ideas are subsequently revealed. We investigate the effects of mixed-anonymity on individually generated brainstorming outputs in two field experiments. The first adopts a between-subject design (N = 216), randomly assigning participants to anonymous, non-anonymous, or mixed-anonymity conditions. The second follows a within-subject design (N = 192), exposing each participant to all three conditions. In both studies, participants engaged in nominal brainstorming tasks. Ideation outcomes were measured by quantity, novelty, and user value. Additionally, we assessed evaluation apprehension and free riding as potential mediators. Our between-subject experiment did not demonstrate significant differences in idea quantity, novelty, or user value among the three anonymity conditions. Conversely, the within-subject experiment revealed significantly higher idea novelty under mixed-anonymity conditions compared to anonymous and non-anonymous conditions, but no significant effects on idea quantity or user value. Contrary to initial hypotheses, the proposed mediators, evaluation apprehension and free riding, did not explain these results. These findings refine the current understanding of anonymity in nominal brainstorming by showing that mixed-anonymity does not yield consistent effects across experimental settings. Overall, this study indicates that the role of mixed-anonymity in individual idea generation is contingent, not as straightforward as existing theorizing based on evaluation apprehension and free riding would suggest.

## Introduction

Generating novel and valuable ideas is a central challenge for organizations aiming to remain innovative in increasingly dynamic and uncertain environments [1]. For this, creativity workshops and creativity techniques play an important role [2].

As firms expand their efforts to leverage employee creativity, structured and comparably easy ideation formats such as brainstorming have gained renewed attention [3,4]. Among these, nominal brainstorming—where individuals generate ideas independently rather than through real-time group interactions—is widely used in research and practice due to its scalability and adaptability across contexts [5,6].

However, the effectiveness of nominal brainstorming highly depends on the social cues embedded in the task design, particularly whether idea creators remain anonymous or are identifiable [7,8]. Anonymous brainstorming has often been found to reduce evaluation apprehension and encourage uninhibited idea sharing [9,10], but may also foster disengagement or free riding due to the absence of personal accountability [11,12]. Conversely, non-anonymous brainstorming can motivate participants through recognition and visibility but may suppress the expression of unconventional or risky ideas [13].

Although anonymity can both reduce evaluation apprehension and encourage free riding, empirical results have been inconsistent [14–16]. We therefore introduce a hybrid approach, mixed-anonymity, in which participants generate ideas independently and anonymously, but the authors of the top ideas are revealed post-evaluation. We hypothesize that this design combines the psychological safety of anonymity with the accountability of non-anonymous formats, thereby improving both the quantity and quality of individually generated ideas. To test this, we conducted two field experiments, one between-subjects and one within-subjects, comparing mixed-anonymity to anonymous and non-anonymous conditions, and we measured evaluation apprehension and free riding as potential mediators.

Research on creativity is of a truly interdisciplinary nature, embedded in different disciplines, such as neuroscience, marketing, education, psychology, sociology, computer science or management science [17]. We embed this research at the intersection of management science and psychology, where we also identified most of our related research.

## Background

Creativity plays a central role in the success of modern organizations. It is widely acknowledged that the ability to generate novel and useful ideas is critical for driving innovation, improving processes, and solving complex problems in dynamic environments [3]. Organizations increasingly face challenges that require innovative solutions, and creativity has become a critical factor in maintaining competitiveness. It is increasingly understood not as a fixed trait but as a contextually modulated process, shaped by social cues and cognitive framing [18]. To foster creativity, companies often rely on structured methods and initiatives, such as innovation contests [19], lead user workshops [20], or idea management systems [21]. These approaches provide employees with opportunities to contribute ideas that can drive technological advancements, optimize operations, or create new business models.

Among the various methods used to stimulate creativity in organizations, brainstorming is one of the most widely adopted techniques. Brainstorming, first introduced by Osborn [22], is an established creativity technique designed to generate a large number of ideas to solve a specific problem. The basic principle is to encourage participants to freely suggest ideas without criticism or immediate evaluation, fostering open and spontaneous thinking [22]. In its traditional group-based form, brainstorming involves a facilitator presenting a problem to a group, after which participants are encouraged to contribute as many ideas as possible, building on others’ suggestions when possible. The collaborative nature of this form of brainstorming fosters a sense of shared ownership [23] and can stimulate creative thinking by exposing individuals to diverse perspectives [4]. Previous research suggests that its popularity in organizational contexts stems from its inclusive nature and potential to facilitate cross-functional idea exchange [24].

While brainstorming can be structured to meet different needs – either through group brainstorming, where participants contribute ideas in real time, or through nominal brainstorming, where individuals generate ideas independently [25–27] – it faces several challenges. In group brainstorming, for example, production blocking may occur when participants wait for their turn to speak, disrupting the flow of ideas [8,28]. Additionally, idea fixation can arise, where individuals become overly focused on existing suggestions, thereby limiting the generation of novel ideas [29]. Another barrier is cognitive inertia, where participants tend to follow the same line of thinking without deviating from the current theme, leading to a lack of diversity in ideas [30,31]. Despite these challenges, brainstorming remains popular in organizations for its flexibility and potential to generate diverse perspectives, which are essential for addressing complex problems.

Although brainstorming is widely employed, research has produced conflicting results regarding its overall effectiveness. Studies have examined various factors that influence brainstorming performance, including group structures [5], trained facilitators [32], task framing [33], and brainstorming instructions [34]. Among these, anonymity has received significant attention as a potential factor influencing participant behavior and idea generation. Some research suggests that anonymity can have a positive effect, such as encouraging participants to contribute more freely without fear of judgment [8,13–16,35]. At the same time, however, conflicting findings have emerged about whether anonymity can also lead to disengagement and a lack of accountability, which can reduce the quality of ideas generated [36]. This tension between the potential benefits and drawbacks of anonymity has resulted in an ambiguous evidence base, leaving open questions about its overall impact on brainstorming performance. The present study addresses this ambiguity in the context of nominal brainstorming. Accordingly, the remainder of the manuscript uses the term brainstorming to refer to this individual form unless otherwise specified.

## Theory

In *anonymous brainstorming*, the absence of personal identifiers helps reduce evaluation apprehension, which is a significant barrier to idea sharing [8,37,38]. Evaluation apprehension arises when individuals hold back ideas due to fear of negative judgment, particularly in settings where contributions are publicly attributed. By removing identifiable attribution, anonymous brainstorming provides participants with a sense of psychological safety, encouraging them to contribute a broader range of ideas without concern for how others might perceive them [14]. This aspect of anonymity can stimulate creative expression and diversity of ideas, as participants feel less inhibited and more willing to share unconventional or bold suggestions.

However, anonymity can also lead to free riding, in which individuals feel less accountable for their contributions and may exert less effort [8,39]. Without identifiable attribution, participants have fewer incentives to fully engage, as their contributions are indistinguishable from others’ [36,40]. This tendency can lead to reduced participation or less rigorous contributions, thereby diminishing the overall quality of the ideas generated. Thus, while anonymity can foster openness, it may also limit accountability, affecting both the quality and quantity of contributions in brainstorming sessions.

In contrast to anonymous settings, *non-anonymous brainstorming* emphasizes accountability by making each participant’s contributions visible to others [8]. This transparency can serve as a motivational factor, as individuals may feel compelled to contribute actively and put more effort into their ideas when their names are associated with their input. By attributing ideas to specific individuals, non-anonymous brainstorming reduces the likelihood of free riding because each person’s input can be directly linked to them [36,40]. As a result, non-anonymous settings may increase participants’ visible engagement due to social pressure and the potential for peers or supervisors to evaluate their contributions.

However, the visibility of contributions in non-anonymous brainstorming can also intensify evaluation apprehension [41]. When participants know their ideas will be directly associated with them, they may hesitate to share more unconventional or risky ideas out of concern for negative judgment [8,13]. This tendency to self-censor can limit creativity, as individuals might favor safer, less innovative suggestions that align with perceived expectations. Thus, while non-anonymity fosters accountability and may reduce free riding, it can simultaneously inhibit the expression of bold or unique ideas due to heightened evaluation apprehension. In summary, both mechanisms could cancel each other out, leading to similar outcomes in both anonymous and non-anonymous brainstorming [14–16].

In this paper, we propose a third form of anonymity that overcomes the drawbacks of both anonymous and non-anonymous brainstorming without sacrificing the benefits: *mixed-anonymity brainstorming*. In this hybrid approach, participants generate ideas anonymously, but only the identities of the creators of the top-rated ideas are revealed after the evaluation phase. This method is designed to minimize evaluation apprehension during idea generation, as participants are initially free to contribute without fearing immediate personal judgment. By ensuring anonymity throughout the brainstorming phase, mixed-anonymity encourages individuals to share a broader range of ideas, potentially including more unconventional or creative suggestions.

At the same time, mixed-anonymity brainstorming introduces accountability by revealing the identities of those whose ideas are highly rated, helping address free riding. The prospect of having their identities disclosed for top ideas can incentivize participants to contribute high-quality ideas, knowing that strong contributions may lead to recognition. This dual structure allows mixed-anonymity to balance the psychological safety of anonymous brainstorming with the motivational benefits of non-anonymous settings. By combining these elements, mixed-anonymity can improve both the quality and quantity of ideas generated in brainstorming sessions.

Based on this reasoning, we propose the following hypotheses:

H1: Due to a lower likelihood of free riding, mixed-anonymity leads to better ideation outcomes than anonymous brainstorming.

H2: Due to a lower likelihood of evaluation apprehension, mixed-anonymity leads to better ideation outcomes than non-anonymous brainstorming.

## Between-subject field experiment

### Materials and methods

#### Design.

We conducted a between-subject field experiment to examine how different anonymity conditions affect participants’ ideation outcomes in a brainstorming task. Participants were randomly assigned to one of three conditions: anonymous brainstorming, non-anonymous brainstorming, or mixed-anonymity brainstorming. This design allowed us to compare the proposed hybrid condition against each of the two conventional anonymity conditions individually and assess whether it combines the benefits of both. In all three conditions, participants generated ideas individually (i.e., nominal brainstorming), a setting that allows for high experimental control while retaining practical relevance for real-world brainstorming contexts.

The conditions differed in how participants were informed their identities would be handled after idea generation:

In the *anonymous condition*, participants were informed that their ideas would be presented to evaluators without their names being revealed and that the creators of the top three ideas would remain anonymous. In the *non-anonymous condition*, participants were told that their ideas would be attributed to them and that the creators of the top three ideas would be publicly named. In the *mixed-anonymity condition*, participants were informed that their ideas would be evaluated anonymously but that the names of the creators of the top three ideas would be disclosed after the evaluation.

These instructions were provided in a standardized written format. After receiving the respective instructions, participants completed an ideation task in which they were asked to generate up to ten ideas in response to a given challenge. Following the idea generation phase, they completed a survey that included measures of the proposed mediators (evaluation apprehension and free riding) and demographic variables. All data were collected before the widespread availability of generative AI tools. The design of this experiment was preregistered (https://www.socialscienceregistry.org/trials/4484).

#### Data collection.

The sample size for the between-subject experiment was guided by an a priori power analysis reported in the preregistered trial protocol. Using G*Power for a three-group ANOVA, we assumed Cohen’s f = 0.25 as the expected effect size. This estimate was used as an approximate planning value based on prior experimental work by Girotra, Terwiesch, and Ulrich [5], who compared different ideation setups in a related brainstorming context. The required sample size was estimated at 252 participants for a power of.95 and 159 participants for a power of.80, assuming α = .05 and balanced group sizes. Based on this calculation, we aimed for approximately 75 participants per condition.

We collected three samples using an identical experimental design but varying the ideation challenge, resulting in an analytic sample of 216 participants who contributed at least one idea and a total of 830 ideas. The first sample comprised 98 participants from an entrepreneurship course at a large German university, contributing 328 ideas. The second sample included 12 participants from two entrepreneurship courses at the same university, contributing 43 ideas. The third sample consisted of 106 participants from a Dutch university enrolled in a course on academic methods, contributing 459 ideas.

During a brainstorming session, participants were asked to generate ideas for new product or service concepts targeting the student market. The ideation tasks followed a standardized structure and were adapted from Girotra, Terwiesch, and Ulrich [5], asking participants to imagine being commissioned by a manufacturer or organization to suggest innovative concepts in a specific domain. While the underlying instruction format remained consistent, the thematic focus of the challenges varied slightly across samples. An example instruction is given below:

*“You have been retained by a manufacturer of food and beverage products to identify new product concepts for the student market. The manufacturer is interested in any product that might be sold to students in a food market. The manufacturer is particularly interested in products likely to be appealing to students. These products might be solutions to unmet needs or improved solutions to existing needs. Please come up with ideas for new product concepts in the field of food and beverage products for the student market.”*

Participants were recruited between 11 January 2022 and 19 February 2024. Across all samples, participation was voluntary and unrelated to academic performance. The students were informed with oral and written instructions and signed consent forms before participating in the study. The study was reviewed and approved by the Ethics Committee of the Faculty of Behavioural, Management and Social Sciences at the University of Twente (approval no. 210748, 04.05.2021) for sample 3. For samples 1 and 2, the identical study design, recruitment, data collection, and analysis procedures were applied. According to institutional and national guidelines, no additional formal ethics approval was required, as the study was non-invasive, involved adult volunteers, did not include minors or vulnerable groups, and posed no potential risk to participants.

#### Variables.

To examine whether mixed-anonymity enhances brainstorming performance compared to anonymous and non-anonymous conditions, we measured ideation outcomes along two dimensions: idea quantity and idea quality. For idea quantity, we counted the number of ideas provided by each individual. Idea quality was assessed in terms of novelty and user value.

For novelty and user value, the ideas were rated by judges on a 7-point Likert scale using the consensual assessment technique [42], which has been used in previous studies [43–45]. As the thematic focus of the ideation challenges varied slightly across samples, the contextual framing in the rating instructions referred to the respective brainstorming task. The rating criteria and scale were kept consistent. An example of the instructions given to the judges is provided below:

*“The following ideas were generated in brainstorming sessions in which participants were asked to develop new product concepts in the field of food and beverage products for the student market. Please review each idea carefully and rate it based on the criteria of novelty and user value. Novelty refers to ‘uniqueness and originality’ and is rated on a scale from 1 (not novel at all) to 7 (very novel). User value refers to ‘the extent of benefit and value for users’ and is rated on a scale from 1 (no value to users) to 7 (great value to users).”*

We then took the average of all raters to build these variables. The judges rated the ideas in random order and were blind to the conditions or the name of the idea creator. It should be noted that this approach is not in line with the rules communicated to participants, but was necessary to ensure that any observed effects reflect the influence of anonymity on participants’ idea generation rather than potential biases in evaluation. Although the experiment focuses on the former aspect, one could argue that judges might adjust their evaluation depending on whether or not the creator’s identity is revealed to them. Participants’ identities were hidden from the judges to overcome this potentially confounding effect.

For the ideas from samples 1 and 2, the judges consisted of three university students, one university lecturer, and five external managers in the respective field of the ideation challenge. Due to their familiarity with the ideas’ context and relevant professional backgrounds, the diverse group was considered suitable to assess the ideas in terms of novelty and user value. For the ideas from sample 3, the judges consisted of seven university students. As members of the target demographic for the ideas, they were considered suitable to evaluate them in terms of novelty and user value. Inter-rater agreement was poor for novelty and moderate for user value in samples 1 and 2, and good for novelty and moderate for user value in sample 3 (samples 1 and 2: novelty ICC(2,9) = 0.361, user value ICC(2,9) = 0.639; sample 3: novelty ICC(2,7) = 0.843, user value ICC(2,7) = 0.537), which is consistent with prior research on subjective idea evaluations. We, therefore, use mean ratings across judges for all analyses. Each idea was evaluated independently by multiple judges, and the analyses rely on aggregated rather than individual evaluations. Prior research has shown that inter-rater agreement in creativity assessment can vary depending on judge composition and task characteristics, even when common rating dimensions are provided [46]. Additionally, rater differences may occur because judges apply shared criteria with different interpretations of the rating scale and the degree of severity in criterion application when evaluating open-ended responses [47]. Accordingly, the aggregated ratings were retained for the analyses, while the variability in inter-rater agreement across samples, and in particular the limited agreement for novelty in Samples 1 and 2, is acknowledged as a measurement limitation.

We adapted established measures from Dennis and Valacich [12] to quantify evaluation apprehension and free riding. Both constructs were assessed using a 7-point Likert scale, ranging from 1 (I fully disagree) to 7 (I fully agree). Evaluation apprehension was captured by items addressing feelings of nervousness, unease, and concern about criticism during idea generation (Cronbach’s α = 0.776). Free riding, in turn, was measured by items reflecting personal engagement, satisfaction with one’s performance, and motivation to generate quality ideas; this scale was reverse-scored so that higher scores indicate greater active participation (Cronbach’s α = 0.781). Detailed item wording is provided in Table 1.

**Table 1:** Scales for measurement.

Evaluation apprehension
I felt apprehensive generating and sharing ideas.
I was not at ease during the idea generation.
I was worried that my ideas would be criticized by the evaluators.
I did not express all of my ideas because I didn’t want the evaluators to think I was weird or crazy.
**Free riding**
I feel I participated a great deal in this idea generation session.
I am satisfied with my own performance on this task.
I was very motivated to generate quality ideas.

### Results

Please find correlations and summary statistics in Table 2.

**Table 2:** Correlation and summary statistics in Experiment 1.

		n	mean	SD	min	max	1	2	3	4	5	6	7
**1**	**Idea quantity**	215	3.809	2.265	0.000	10.000	1.000						
**2**	**Novelty**	215	3.171	1.025	1.000	6.286	−0.228	1.000					
**3**	**User value**	215	4.106	0.780	1.750	5.750	0.069	0.193	1.000				
**4**	**Mean novelty**	215	3.291	0.811	1.000	5.500	−0.181	0.719	0.060	1.000			
**5**	**Mean user value**	215	4.073	0.610	1.889	5.214	0.009	0.062	0.651	0.140	1.000		
**6**	**Free riding**	215	3.589	1.233	1.000	7.000	−0.280	0.018	−0.117	−0.061	−0.036	1.000	
**7**	**Evaluation apprehension**	215	2.808	1.301	1.000	6.500	−0.131	0.001	0.106	−0.111	−0.021	0.335	1.000

A linear regression analysis was conducted to examine the effects of different anonymity conditions on the number of ideas generated at the participant level (n = 215), as shown in Table 3. Participants in the anonymous condition generated, on average, 0.355 fewer ideas than those in the mixed-anonymity condition (p = 0.431, Cohen’s D = −0.129), and participants in the non-anonymous condition generated 0.283 fewer ideas (p = 0.455, Cohen’s D = −0.123), but neither difference was statistically significant. This suggests no strong evidence of a detrimental effect from these conditions on idea generation compared to the mixed-anonymity condition. The mixed-anonymity condition does not perform significantly better than the anonymous or the non-anonymous condition.

**Table 3:** Regression results for idea quantity and quality in Experiment 1.

	(1)	(2)	(3)	(4)	(5)	(6)	(7)	(8)	(9)	(10)	(11)
	Idea quantity	Novelty	User value	Idea quantity	Mean novelty	Mean user value	Idea quantity	Mean novelty	Mean user value	Free riding	Evaluation apprehension
**Anonymous** ^**1**^	−0.355	0.075	0.147							0.094	−0.219
	(0.366)	(0.170)	(0.093)							(0.191)	(0.205)
**Non-anonymous** ^**1**^	−0.283	0.028	0.075							−0.060	0.193
	(0.372)	(0.139)	(0.097)							(0.194)	(0.209)
**Sample 2** ^**2**^	0.180	−0.356*	0.072	1.246+	−0.088	0.057	0.613	−0.129	0.097	1.829***	1.193**
	(0.691)	(0.154)	(0.108)	(0.693)	(0.252)	(0.185)	(0.678)	(0.241)	(0.176)	(0.360)	(0.387)
**Sample 3** ^**2**^	0.976**	−0.622***	0.410***	0.991**	−0.424***	0.422***	1.197***	−0.403***	0.448***	−0.005	0.645***
	(0.314)	(0.120)	(0.082)	(0.299)	(0.109)	(0.080)	(0.314)	(0.112)	(0.082)	(0.164)	(0.176)
**Free riding**				−0.541***	−0.042	−0.003					
				(0.127)	(0.046)	(0.034)					
**Evaluation** **apprehension**							−0.347**	−0.034	−0.043		
							(0.120)	(0.043)	(0.031)		
**Constant**	3.551***	3.539***	3.781***	5.211***	3.663***	3.883***	4.194***	3.602***	3.976***	3.445***	2.419***
	(0.304)	(0.096)	(0.082)	(0.488)	(0.177)	(0.130)	(0.365)	(0.130)	(0.095)	(0.159)	(0.171)
**n**	215	830	830	215	215	215	215	215	215	215	215
**P**	0	0	0	0	0	0	0	0	0	0	0

Standard errors in parentheses.

+ *p* < 0.10, * *p* < 0.05, ** *p* < 0.01, *** *p* < 0.001.

^1^Reference: Mixed-anonymity.

^2^Reference: Sample 1.

Next, we turn to novelty of ideas. A linear regression analysis on idea level (n = 830) was conducted to assess the impact of different conditions on the novelty of ideas among participants. Because participants contributed multiple ideas, observations at the idea level are not independent within participants. We therefore adjusted standard errors for clustering by participants to account for this nested data structure. The coefficient for participants in the anonymous condition was 0.075 with a p-value of 0.660 (Cohen’s D = 0.069), indicating that this effect was not statistically significant. The effect in the non-anonymous condition was even smaller, with a coefficient of 0.028. The p-value of 0.842 indicates no significant impact of this condition on novelty (Cohen’s D = 0.026). This suggests no strong evidence of a detrimental effect from these conditions on idea generation compared to the mixed-anonymity condition. The mixed-anonymity condition does not produce significantly more novel ideas than the anonymous or non-anonymous conditions.

Next, we turn to user value of ideas on idea level (n = 830), running the same analysis as for novelty. Participants in the anonymous condition were associated with a coefficient of 0.147 for perceived user value and a p-value of 0.115 (Cohen’s D = 0.182), suggesting that this effect is not statistically significant. The coefficient for the non-anonymous condition was 0.075 with a p-value of 0.440 (Cohen’s D = 0.093). This suggests that being in the non-anonymous condition does not significantly influence the perceived user value of ideas. The mixed-anonymity condition does not produce significantly more useful ideas than the anonymous or non-anonymous conditions.

In sum, we have to reject both H1, which predicted that mixed-anonymity leads to better ideation outcomes than anonymous brainstorming due to lower free riding, and H2, which predicted that mixed-anonymity leads to better ideation performance than non-anonymous brainstorming due to a lower likelihood of evaluation apprehension.

There could be various reasons why we did not find the expected results: First, the mediators may have been meaningful, but the condition did not influence them as expected. Second, the condition may have affected the mediators, but the mediators did not impact ideation outcomes. Third, the condition did not affect the mediators, and the mediators did not influence ideation outcomes either.

To understand why our predictions failed, we first want to examine whether our mediators affected ideation outcomes as expected. To test this, we use specifications similar to those in the regressions above but with the mediators as independent variables. To do so, we created the means of idea novelty and user value per person.

We start by regressing ideation outcomes on free riding. We find that free riding is significantly related to the number of ideas (β = −0.541, p = 0.000) but is unrelated to idea novelty (β = −0.042, p = 0.290) and user value (β = −0.003, p = 0.772). Next, we regress free riding on our anonymity conditions. Contrary to our expectations, we do not find that mixed-anonymity is associated with less free riding than anonymous brainstorming. Anonymous brainstorming is associated with more free riding compared to mixed-anonymity (β = 0.094, p = 0.624), but this is not significant. The non-anonymous condition is negatively but not significantly different from mixed-anonymity (β = −0.060, p = 0.757).

We continue by regressing ideation outcomes on evaluation apprehension. We find that evaluation apprehension is significantly related to the number of ideas (β = −0.347, p = 0.005) but is unrelated to idea novelty (β = −0.034, p = 0.410) and user value (β = −0.043, p = 0.164). Next, we regress evaluation apprehension on our anonymity conditions. Contrary to our expectations, we do not find that mixed-anonymity is associated with less evaluation apprehension than non-anonymous brainstorming. Non-anonymous brainstorming is associated with more evaluation apprehension compared to mixed-anonymity (β = 0.193, p = 0.433), but this is not significant. The anonymous condition is lower but not significantly different from mixed-anonymity (β = −0.219, p = 0.255).

## Within-subject field experiment

### Materials and methods

#### Design.

To support the results of the between-subject design, we conducted another experiment in a within-subject design, similar to Girotra et al. [5]. All participants are exposed to every condition of the three between-subject anonymity conditions, with the order of exposure fully balanced to reduce possible positional effects [48]. During each session, three different but comparable brainstorming challenges are presented to all participants in random order. This procedure was intended to reduce possible carry-over effects.

Through this second experimental approach, the values of the dependent variable are determined for all participants in all conditions. Consequently, by comparing the dependent variable in each experimental condition within the same person, the experimental effect of the independent variable on the dependent variable becomes apparent. As each participant represents their own control person, person-related confounding variables are reduced, and the existing residual variance is relatively small [49].

#### Data collection.

The sample included students recruited from a large Dutch University in an innovation course, recruited between 8 September 2021 and 22 September 2022. Participation was voluntary and not linked to academic performance, but students were asked to take part in an experiment as part of a research project. The students were informed with oral and written instructions and signed consent forms before participating in the study. The study was reviewed and approved by the Ethics Committee of the Faculty of Behavioural, Management and Social Sciences at the University of Twente (approval no. 210748, 04.05.2021). In this sample, 192 students contributed at least one idea, providing 2225 ideas across the three rounds.

#### Variables.

All variables except idea novelty and user value were measured as in the between-subject field experiment. In contrast, idea novelty and user value were assessed using GPT-4, an advanced generative AI model developed by OpenAI and accessed via the ChatGPT service. Recent research suggests generative AI closely approximates human decision-making, thereby providing a reliable alternative for assessing subjective criteria [50–52]. In addition, we draw on a separate validation study in which GPT-4 ratings were directly compared with human evaluations using an independent sample of 458 ideas generated in an electronic brainstorming task [53]. Using identical task contexts and rating instructions for human and AI evaluators, this study shows strong correspondence between GPT-4 and human judgments, particularly for novelty (r = 0.883) and purchase intent (r = 0.732), and moderate correspondence for user value (r = 0.506), with all correlations significant at p < .001. Regression analyses further demonstrate substantial predictive power of GPT-4 ratings for human scores (R² up to 0.784). While GPT-4 evaluations were systematically higher in absolute terms, the relative ranking of ideas closely mirrored human assessments. These findings support the validity of using GPT-4 to assess idea novelty and user value in the present study. To ensure procedural consistency, the same prompt structure, evaluation criteria, and response format were used across all idea evaluations. An example of the prompt we used to rate the ideas is given below:

*“The following ideas are the outcome of a brainstorming session where students received the following challenge:*

*<<You have been retained by a manufacturer of food and beverage products to identify new product concepts for the student market. The manufacturer is interested in any product that might be sold to students in a food market. The manufacturer is particularly interested in products likely to be appealing to students. These products might be solutions to unmet needs or improved solutions to existing needs. Please come up with ideas for new product concepts in the field of food and beverage products for the student market.>>*

*Now, please evaluate the following idea that is an outcome of that brainstorming in terms of novelty (Uniqueness and Originality on a scale from 1 (not novel at all) to 7 (very novel)) and user value (The extent of benefit and value for users from 1 (no value to users) to 7 (great value to users)).*

*Please export the findings as Excel.”*

### Results

Please find correlations and summary statistics in Table 4.

**Table 4:** Correlation and summary statistics in Experiment 2.

		n	mean	SD	min	max	1	2	3	4	5	6	7
**1**	**Idea quantity**	189	5.762	2.728	0.000	10.000	1.000						
**2**	**Novelty**	2225	3.899	1.998	1.000	7.000	−0.023	1.000					
**3**	**User value**	2225	3.987	1.980	1.000	7.000	−0.053	0.033	1.000				
**4**	**Novelty mean**	189	3.897	1.120	1.000	7.000	−0.041	0.553	0.026	1.000			
**5**	**User value mean**	189	3.926	1.089	1.000	7.000	−0.096	0.026	0.550	0.048	1.000		
**6**	**Free riding**	189	3.016	1.072	1.000	6.500	−0.403	0.010	0.072	0.018	0.131	1.000	
**7**	**Evaluation apprehension**	189	2.724	1.209	1.000	6.250	−0.171	0.059	0.053	0.107	0.096	0.438	1.000

A linear regression analysis was conducted to examine the effects of different conditions on the number of ideas generated on individual-round level (n = 189), as shown in Table 5. We introduced cross-level fixed effects to control for the contexts and rounds. We do not find a significant difference between the anonymous condition (β = 0.157, p = 0.194, Cohen’s D = 0.130) and the mixed-anonymity condition, nor a difference between the non-anonymous condition (β = −0.099, p = 0.362, Cohen’s D = −0.142) and the mixed-anonymity condition.

**Table 5:** Regression results for idea quantity and quality in Experiment 2.

	(1)	(2)	(3)	(4)	(5)	(6)	(7)	(8)	(9)	(10)	(11)
	Idea quantity	Novelty	User value	Idea quantity	Mean novelty	Mean user value	Idea quantity	Mean novelty	Mean user value	Free riding	Evaluation apprehension
**Anonymous** ^**1**^	0.157	−0.426^***^	0.055							−0.405^*^	−0.601^**^
	(0.113)	(0.114)	(0.108)							(0.203)	(0.217)
**Non-anonymous** ^**1**^	−0.099	−0.275^*^	−0.047							−0.143	−0.400^+^
	(0.108)	(0.109)	(0.114)							(0.180)	(0.215)
**Free riding**				−0.712^***^	0.022	0.126					
				(0.165)	(0.087)	(0.097)					
**Evaluation apprehension**							−0.276^*^	0.116	0.141^+^		
							(0.129)	(0.082)	(0.080)		
**Constant**	3.802^***^	4.345^***^	4.081^***^	5.626^***^	4.013^***^	3.602^***^	4.095^***^	3.780^***^	3.634^***^	3.400^***^	3.142^***^
	(0.183)	(0.183)	(0.212)	(0.628)	(0.319)	(0.325)	(0.483)	(0.262)	(0.258)	(0.135)	(0.148)
**Idea Order FEs**	N	Y	Y	N	N	N	N	N	N	N	N
**Context FEs**	Y	Y	Y	Y	Y	Y	Y	Y	Y	N	N
**Round x Context FEs**	N	Y	Y	N	N	N	N	N	N	N	N
**n**	189	2225	2225	189	189	189	189	189	189	189	189
**P**	0.151	0	1	0	0	1	0	0	0	0	0

Standard errors in parentheses.

+ *p* < 0.10, * *p* < 0.05, ** *p* < 0.01, *** *p* < 0.001.

^1^Reference: Mixed-anonymity.

Next, we turn to novelty of ideas. A fixed-effects regression with standard errors clustered by participant was conducted at the idea level (n = 2225) to account for the nested structure of multiple ideas within participants. We find that the anonymous condition is associated with substantially and statistically significantly lower idea novelty (β = −0.426, p = 0.000, Cohen’s D = −0.224) than the mixed-anonymity condition. The same is true for the non-anonymous condition (β = −0.275, p = 0.013, Cohen’s D = −0.144).

Next, we turn to user value of ideas. A fixed-effects regression with standard errors clustered by participant was conducted at the idea level (n = 2225). We do not find a significant difference between the anonymous condition (β = 0.055, p = 0.611, Cohen’s D = 0.030) and the mixed-anonymity condition, nor a difference between the non-anonymous condition (β = −0.047, p = 0.680, Cohen’s D = −0.025) and the mixed-anonymity condition.

To understand why our predictions fell short, we first need to assess whether our mediators influenced the ideation outcomes as anticipated. To do this, we apply similar specifications used in the previous regressions, but now treat the mediators as independent variables. We only used the last round of evaluation in this analysis since we only solicited the mediators for that round. We also created the mean of idea novelty and user value per person across all ideas (n = 189).

We begin by regressing ideation outcomes on free riding. Our results show that free riding is significantly associated with the number of ideas (β = −0.712, p = 0.000) but has no significant relationship with idea novelty (β = 0.022, p = 0.801) or user value (β = 0.126, p = 0.197). Following this, we regress free riding on our anonymity conditions. Contrary to our expectations, we do not find that mixed-anonymity reduces free riding compared to anonymous brainstorming. In fact, anonymous brainstorming is associated with significantly less free riding than mixed-anonymity (β = −0.405, p = 0.049). The non-anonymous condition, while negatively related to free riding, does not significantly differ from mixed-anonymity (β = −0.143, p = 0.389). Next, we regress ideation outcomes on evaluation apprehension. We find that evaluation apprehension is significantly related to the number of ideas (β = −0.276, p = 0.033), but it does not significantly affect idea novelty (β = 0.116, p = 0.161) or user value (β = 0.141, p = 0.080). We then regress evaluation apprehension on our anonymity conditions. Contrary to our expectations, mixed-anonymity does not result in less evaluation apprehension than non-anonymous brainstorming. Instead, non-anonymous brainstorming is associated with lower evaluation apprehension compared to mixed-anonymity (β = −0.400, p = 0.063), though this effect is not significant. However, the anonymous condition is associated with significantly lower evaluation apprehension compared to mixed-anonymity (β = −0.601, p = 0.005).

## Discussion

In this study, we explored the effects of mixed-anonymity brainstorming on idea generation, expecting it to reduce free riding compared to anonymous brainstorming [39] and evaluation apprehension compared to non-anonymous brainstorming [8,14]. Across the two experiments in the field, however, the pattern of findings was more mixed. In our between-subject field experiment, we found no statistically significant differences in idea quantity, novelty, or user value between mixed-anonymity and the two other anonymity conditions. By contrast, in the within-subject experiment, we observed that the mixed-anonymity condition was associated with significantly higher idea novelty compared to anonymous and non-anonymous settings, while effects on the number of generated ideas and user value remained insignificant. Furthermore, mixed-anonymity did not significantly reduce free riding or evaluation apprehension compared to the anonymous and non-anonymous conditions in either the between-subject or within-subject designs. Taken together, these findings suggest that the only observed statistically significant difference involving mixed-anonymity was higher novelty in the within-subject design.

Our theoretical argument for mixed-anonymity brainstorming drew on two established lines of research: first, that anonymity can reduce evaluation apprehension in idea generation contexts [8,14], and second, that identifiability can shape motivation and thereby reduce tendencies toward free riding [39]. The present findings do not provide support for the claim that mixed-anonymity improves brainstorming performance through this combination of mechanisms. Although mixed-anonymity was associated with higher novelty in the within-subject experiment, this pattern was not accompanied by corresponding differences in evaluation apprehension or free riding, and no comparable pattern emerged in the between-subject experiment. Accordingly, the data do not show that mixed-anonymity enhanced brainstorming performance through the mechanism underlying our hypotheses.

These null results for the proposed mediators are consistent with a broader pattern in research on identity disclosure. Studies examining the effects of making contributor identities visible have found that disclosure can shift the distribution of contribution quality without necessarily changing average output levels, and that these effects are explained less by apprehension or effort withdrawal than by self-presentational concerns [41]. From this perspective, the mixed-anonymity design examined here combines two conceptually distinct features: anonymity during the generation phase, which shields contributors from immediate social evaluation, and selective ex-post recognition, which introduces the prospect of status gain for the authors of top-rated ideas. These two components are not equivalent to a simple combination of anonymous and non-anonymous conditions, and they may activate different behavioral responses: A protective motive to avoid reputational risk during generation and an acquisitive motive to pursue recognition through high-quality contributions after evaluation. The present study did not measure these self-presentational processes directly; therefore, it is not possible to determine whether they were activated or accounted for the novelty difference observed in the within-subject experiment. This represents a meaningful direction for future research.

Despite not finding what we expected, the present study makes a contribution to research on anonymity in brainstorming by introducing and empirically testing mixed-anonymity as a distinct condition that moves beyond the usual dichotomy between anonymous and non-anonymous brainstorming. More precisely, the design examined here instantiates a specific disclosure policy: idea generation remains anonymous while the identities of only the highest-rated contributors are revealed after evaluation. This structure (varying the timing and selectivity of identity disclosure rather than simply toggling anonymity on or off) has not previously been examined in ideation research, and it connects to the broader brainstorming literature [e.g., 4]. Furthermore, the findings suggest that the effects of anonymity conditions may be more context-dependent than our theoretical argument and hypotheses implied. More specifically, mixed-anonymity was associated with higher novelty only in the within-subject experiment, whereas no corresponding differences emerged in the between-subject experiment. The contribution of the present study, therefore, lies less in confirming the explanatory logic underlying H1 and H2 than in identifying a specific empirical pattern that is not fully explained by the current theoretical account. In this sense, the study helps refine the research agenda by identifying a potentially relevant phenomenon while also delimiting the explanatory reach of the current theory.

A further observation that warrants attention concerns the outcome dimension on which the effect emerged. The significant difference in the within-subject experiment was specific to idea novelty and did not extend to idea quantity or user value. This selective pattern is consistent with research suggesting that disclosure policies may shift the distributional properties of contributions, particularly the upper tail, rather than uniformly raising average output [5]. Organizations rarely optimize for average ideas; they typically select from a small number of highly rated submissions. It is therefore possible that the mixed-anonymity condition examined here had a stronger effect on the quality of the best ideas generated per participant than on mean novelty across all ideas. The present analyses were not designed to test this specifically, but future research could examine whether selective ex-post disclosure affects the right tail of the idea quality distribution — for instance, by analyzing the most novel idea per participant or quantile treatment effects — rather than relying solely on mean comparisons.

The significantly higher novelty observed in the within-subject design should therefore be interpreted cautiously. Because participants in Experiment 2 experienced all anonymity conditions, the design may have increased the salience of the manipulation and, with it, participants’ ability to infer the study purpose. Such demand characteristics can alter participant behavior when individuals adjust their responses in light of perceived expectations rather than the treatment itself [54,55]. In addition, within-subject designs are generally more susceptible than between-subject designs to carryover and contextual comparison effects, because responses in a given condition may be shaped by prior exposure to other conditions rather than by that condition alone [56]. Even when condition order is counterbalanced, serial order and carryover effects cannot be ruled out entirely, as responses may remain dependent on preceding states or experiences [57]. Finally, repeated exposure across conditions may also increase sensitivity to subtle procedural or experimenter-related cues, which can influence participant behavior independently of the focal manipulation [58]. The present design does not allow these alternatives to be disentangled from a possible effect of the mixed-anonymity condition. This caution is particularly relevant because the observed difference was limited to novelty and did not extend to idea quantity or user value. Accordingly, the higher novelty observed in the within-subject experiment should be interpreted as a design-specific pattern that requires replication under conditions that more clearly isolate causal effects.

Other limitations should be considered when interpreting the findings of the study. First, the finding that mixed-anonymity was associated with higher novelty in the within-subject experiment cannot be cleanly separated from design-specific influences such as demand characteristics, contextual comparison, or other carryover-related processes. Second, the measurement of idea quality in Experiment 1 was subject to limited inter-rater agreement, particularly for novelty in samples 1 and 2, which reduces confidence in the precision of the human-coded outcome measures. Although some variability in subjective creativity ratings is common because judges may differ in their use of criteria and rating scales, this measurement limitation should be considered when interpreting the findings [47,59]. Future research should therefore seek to replicate the within-subject finding under conditions that more clearly isolate treatment effects and should further strengthen the assessment of idea quality through more robust rating procedures, improved rater training, or multiple convergent evaluation approaches. Another starting point for further research is the use of alternative methods for creativity. Here, the field of psychology offers many starting points. Recently, especially in healthcare, many different approaches have gained momentum at both the individual and group levels. A prominent example is concept mapping, in which individual and group creativity are combined with further statistical analysis [27,60–62]. Or group concept mapping, where stakeholder engagement is organized around a specific topic. To apply such methods and then compare these results with the ones from our study will surely lead to interesting insights. Also, thinking of generative Artificial Intelligence Tools could lead to new forms of creativity workshops [63]. The same is true for physical spaces and educational background, and their influence on creativity [64,65].

Finally, our three samples were drawn from two universities: two samples from a German university and one from a university in the Dutch-German border region, but we did not collect individual-level demographic data. This prevents formal verification of sample comparability and precludes a post-randomization balance table. We note that the border-region university draws a student population with strong cultural and institutional overlap with the German samples, but we acknowledge that this contextual argument cannot substitute for empirical evidence. Future studies should collect and report demographic characteristics across sites. Second, site was not modeled as a random effect; instead, the sample was included as a fixed covariate in all regressions. This controls for additive site differences but does not account for potential site-by-condition interactions. Replication across more diverse institutional contexts would strengthen the generalizability of our findings.

From a practical perspective, the present findings suggest that the sequencing of anonymity and identifiability may merit closer attention when organizations design brainstorming processes. More specifically, the mixed-anonymity condition examined in this study, in which idea generation remains anonymous while selected contributors are identified only after evaluation, may be worth pilot-testing in ideation settings where novelty is a central objective. Because this pattern was observed only in the within-subject experiment, this practical implication should be interpreted as preliminary and context-dependent. In practice, this could mean selectively implementing mixed-anonymity settings for brainstorming sessions that are focused on breakthrough innovation, where unconventional ideas are encouraged and rewarded. Additionally, organizations might use mixed-anonymity alongside other formats, allowing employees to experience different levels of anonymity across sessions. This approach could help participants find their comfort zone for ideation, potentially maximizing both novelty and engagement across different participants. But as mentioned earlier, we can only rely on preliminary results, so much more research is necessary to have more empirical evidence.

## Supporting information

S1 File Raw data from the between-subject field experiment. (CSV)

S2 File Raw data from the within-subject field experiment. (CSV)
